# "Mad Doctors"—A Protest

**Published:** 1891-08-15

**Authors:** A. R. Urquhart

**Affiliations:** Physician Superintendent, James Murray's Royal Asylum, Perth


### "Mad Doctors"—A Protest.

By A. R. Urquhart, M.D., Physician Superintendent, James Murray's Royal Asylum, Perth.

I read your annotation entitled "Sir Charles Russell and the Mad Doctors" with great disfavour. Do you, as responsible for the opinions of The Hospital, indeed maintain these views? or is the editor on holiday? The Hospital circulates in this institution week after week in the hope of impressing all concerned that this, too, is an hospital with the intention of curing the curable and relieving the hopeless. It is not seemly that such a heading should be found on the pages of a journal designed to aid "science, medicine, nursing, and philanthropy."

The use of the offensive title is, perhaps, a matter of taste; and it is not my purpose to further notice a personal affront. The main object of my writing is to controvert in the strongest manner your statement that a patient ought not to be placed in an asylum unless he is "dangerous." Such a statement is neither scientific, medical, nor philanthropic. Do you suppose that men in my position are content to be mere jailors? The asylum physician is first of all a physician, and the question for the medical profession is not whether A is dangerous to himself or others, but whether it is to A's best interest that he should be treated medically in an asylum.

Furthermore, the law of England is brand new. It was to be a perfect panacea for all the troubles of the world of lunacy. It was a Consolidation Act. It was specially designed to enable the magistrate to try the lunatic. The opinions of those who had long and intimate experience in lunacy practice were unheeded, and their consequent dissatisfaction is by no means confined to the decent obscurity of asylum reports. The law was constructed to favour the views you now express. It does not become you to find fault.

Then you seek a definition of sanity, and charge medical specialists with chaotic and illogical opinions. It is not in the power of man to define sanity. Chaotic and illogical opinions abound in the religious world, with an open Bible, and Baptists and Pædo-Baptists confidently sure that the other is wrong. The aim of the physician is to ascertain, to predict and to treat, and it is no shame when he does not go beyond stating an opinion founded on experience. He says, "Under certain circumstances I believe that such and such is the best course," and if he says no more, The Hospital should be the last to urge him to the bombast of the charlatan.

Any stick is good enough to beat the madhouse keeper with. There is no reference in your annotation to the difficulties and impossibilities. The pathological changes in a case of general paralysis are now a fixed quantity, and the future of the individual can be predicted with almost unfailing certainty, but the indefinite changes in the brain of a "slight" case of insanity leave ground for much debate and qualified opinion.

Lord Bramwell it was who claimed to be able to diagnose insanity. The subject is matter for never-ending debate between lawyers and doctors, and certainly the doctors will not admit that the lawyers have the best of the argument. The Brisish jury will take the opinion of any expert except that of a physician who has spent his years in the study of insanity. As all men are heaven-born politicians, so is each the infallible judge of his neighbour's mental soundness. When the world asks for practical guidance it follows its rule in not taking the advice tendered. It rather pins its faith on the fortuitous collection of uninformed mediocrity swept into the jury box to be the sport of the most expensive barrister available.

[The expression "Mad doctors" to which our correspondent takes exception was not used with any offensive intention, but simply as a convenient and familiar form of words which everyone would readily understand. We willingly withdraw it. So far as the general opinions expressed in the annotation complained of are concerned, we stand by them, only expressing the conviction that our correspondent has not taken them up rightly. Our contention is that lunacy should be more precisely defined; that there should be classes of lunatics and degrees in lunacy for purposes of administration, even though it may be impossible to make definition and classification so precise as to satisfy scientific purism. We maintain that deprivation of liberty should sometimes be only partial, and sometimes complete. There are certain insane persons who are rightly allowed to be at large. That an asylum is an hospital we gladly admit, but even at an hospital there are partial patients as well as full patients; that is, out-patients as well as in-patients. There is, moreover, a large class of persons who, if tested by a strict standard of health, would be declared to be not well, but who yet are neither in-patients, out-patients, nor patients at all. We must still insist that lunacy specialists have not brought up their work to a standard which makes administration at once easy, just, and satisfactory to the common-sense of the public.—Editor.]

